# “Antibiotics kill things very quickly” - consumers’ perspectives on non-prescribed antibiotic use in Saudi Arabia

**DOI:** 10.1186/s12889-018-6088-z

**Published:** 2018-10-16

**Authors:** Faten Alhomoud, Zainab Aljamea, Lama Basalelah

**Affiliations:** 1 Department of Pharmacy Practice, College of Clinical Pharmacy, Imam Abdulrahman Bin Faisal University, Dammam, 31952 Saudi Arabia; 2 College of Clinical Pharmacy, Imam Abdulrahman Bin Faisal University, Dammam, Saudi Arabia

**Keywords:** Self-medication, Self-treatment, Self-prescription, Non-prescription, Antibiotic, Antibacterial, Saudi Arabia

## Abstract

**Background:**

In recent decades, the Kingdom of Saudi Arabia has seen an exponentially growing antibiotic resistance, which is exacerbated by the use of antibiotics without a prescription and other various factors. However, no published data are available on factors influencing non-prescription use of antibiotics among the general public in Saudi Arabia using an in-depth interview technique.

**Methods:**

Semi-structured interviews were carried out with 40 Saudi participants from the Eastern Province of Saudi Arabia, selected via snowball sampling technique. Participants were enrolled based on the following inclusion criteria: 18 years of age or older and had self-medicated themselves with antibiotics in the past two years. Data collection was continued until data saturation was attained. Interviews were audiotaped, transcribed verbatim and analysed using NVivo 10 software.

**Results:**

Participants (80% female) had a mean (SD) age of 30 years (10.2). Self-medication with antibiotics was associated with various inappropriate antibiotic use behaviours and negative outcomes such as antibiotic resistance, treatment failures and adverse events. Interviews revealed that different reasons contribute to the rise of self-medication with antibiotics, ranging from difficulty accessing healthcare services, participant’s cultural beliefs and practices, lack of knowledge about antibiotics and antibiotic resistance, and weak regulatory enforcement.

**Conclusions:**

The findings of the present study will aid in generating data that may provide an insight when designing future interventions to promote public health awareness regarding safe and effective use of antibiotics.

**Electronic supplementary material:**

The online version of this article (10.1186/s12889-018-6088-z) contains supplementary material, which is available to authorized users.

## Background

Although antibiotics represent one of the major improvements in public health, their misuse and overuse, as a result of self-medication and irrational prescribing and dispensing, may lead to an increased risk of antibiotic resistance [1–3]. Self-medication includes the use of medicinal products by the consumer to treat self-recognised symptoms or disorders [4]. Medicines intended for self-medication are called over-the-counter medicines and are available without a medical prescription. In Saudi Arabia, for example, the accessibility of antibiotics over-the-counter (OTC) without prescriptions is an important factor that contributes to the public’s behaviour in respect of self-medicating [5]. Findings from a study conducted in 2010 in Riyadh, Saudi Arabia, showed that antibiotics could readily be obtained without a prescription in 244 (78%) of 327 pharmacies [6].

Inappropriate self-medication practices with antibiotics may include insufficient dosage and/or frequency, the antibiotic spectrum being either too narrow or too broad, delayed antibiotic therapy in critical patients, unnecessary use of antibiotic for the wrong ailments, and receiving antibiotics from different sources such as patient’s stock, friends and family, without a medical consultation. Patients/consumers may mistakenly make self-diagnosis, with consequences such as failure to recognise symptoms that may be an indication for another disease, and failure to treat the disease effectively. In addition, when a bacterial infection is not presented, patients can expose themselves to the risks associated with antibiotics. Inappropriate utilisation of antibiotics can also lead to increased healthcare costs, poor health outcomes and increased risk of antibiotic resistance, adverse drug reactions and contraindications [7, 8].

Although self-medication with antibiotics (SMA) is common in developing countries, with an overall prevalence of 38.8% (95% CI: 29.5–48.1) [9], semi-developed countries, like Gulf countries, are not immune [3]. For example, a systematic review that included 11 various Middle Eastern countries reported that rates of antibiotic self-medication ranged from 19 to 82% [3]. The attitude towards self-medication appears to differ between Middle Eastern countries, with Gulf countries having much higher rates of self-medication compared to other Middle Eastern countries. In Saudi Arabia, for example, self-medication with antibiotics was found to range from 48 to 79% [3].

Self-medication with antibiotics is a major concern to the Ministry of Health (MOH) and public health sector in Saudi Arabia, a country whose rate of both antibiotic resistance and consumption is one of the highest in the Middle East [3, 10, 11]. Based on a review of the studies conducted in Saudi Arabia from 1990 until 2011, the prevalence of resistant bacteria ranges from 7.6 to 92.3% among gram-negative bacteria with *Pseudomonas aeruginosa*, *Acinetobacter species* and *Klebsiella pneumoniae* having the highest prevalence. Among the gram-positive bacteria, the prevalence of resistance ranges from 23.5 to 30.7%, with *Streptococcus pneumoniae*, *Clostridium Difficile* and Methicillin-resistant *Staphylococcus aureus* (MRSA) showing the highest prevalence [10].

A World Health Organisation (WHO) ([12]) report also shows extensive antibiotic resistance across the WHO Eastern Mediterranean Region. In particular, there are high levels of *Escherichia coli* resistance to third-generation cephalosporins and fluoroquinolones – two important and commonly used types of antibacterial medicine. Resistance to third-generation cephalosporins in *Klebsiella pneumoniae* is also high and widespread. In some parts of the region, more than half of *Staphylococcus aureus* infections are reported to be methicillin-resistant (MRSA), meaning that treatment with standard antibiotics does not work. Even globally, the WHO report reveals that a serious threat is occurring in every region of the world as regards antibiotic resistance and it has the potential to affect anyone, of any age, in any country [12]. In 2013, the Centers for Disease Control and Prevention (CDC) estimated that more than 2 million illnesses and 23,000 deaths were caused by antibiotic resistance [8].

Understanding the Saudi cultural determinants of or reasons for self-medication, and the practices, behaviours and knowledge about antibiotics represents a vital pre-requisite to design and implement effective public health interventions. Self-medication with antibiotics in Saudi Arabia is an issue that requires further investigations or studies using an in-depth interview technique.

### Aim of the study

The current study aimed to explore factors influencing self-medication with antibiotics practice and to give a comprehensive understanding of such practices and the circumstances in which they occur in order to promote better public health awareness of safe and effective antibiotic use.

## Methods

### Study setting, recruitment and data collection

The study population consisted of people who were 18 years of age or older, had self-medicated themselves with antibiotics in the past 2 years, and were able to speak English or Arabic. Individuals located in the Eastern Province of Saudi Arabia were selected and recruited by means of snowball sampling, a purposive sampling method that identified potential subjects by asking other participants to locate individuals with experience and understanding of the SMA phenomenon [13]. Students from the College of Clinical Pharmacy at Imam Abdulrahman Bin Faisal University recommended potential participants from their close family members and relatives, friends and neighbours, who met the inclusion criteria, to take part in the current study. Those participants then recommend additional participants who met the inclusion criteria, and so on, thus building up like a snowball rolling down a hill. Snowball sampling can be used to ease data collection and to recruit people who are difficult to identify or have to meet certain criteria to participate [13].

Individuals were invited by telephone or by sending an invitation letter via email. Once located and informed of the study, all potential subjects were approached and invited to participate. If they were willing to participate, an interview was conducted either face-to-face in participants’ homes or on the telephone, according to the participant’s preference, at a mutually agreeable time. The data collection was conducted during the period from January to June 2017.

A semi-structured interview schedule was developed (see Additional file 1) after undertaking a literature review in the Middle Eastern countries to identify extent and reasons behind self-medication. The interview schedule consists of three parts; the first part requests some personal information such as age, gender, nationality, living arrangements, health insurance, family income per month and educational level. The second part is constructed of 10 close-ended questions intended to assess participants’ knowledge with regards to the antibiotics. This part was assessed out of 10. Participants scoring ≥8 were considered to have good knowledge, those scoring 5–7 were considered to have intermediate knowledge, whereas participants scoring ≤4 were considered to have poor knowledge. The last part contains eight open-ended questions to enable exploration of the participants’ own views regarding self-medication with antibiotics, their perceptions and explanations of how self-medication might arise, and for them to describe factors that may influence this practice.

A patient information sheet was provided to all eligible participants who wished to take part. Informed verbal and written consent was obtained prior to commencing the interviews. The participants were reminded that they could withdraw from the study at any time without providing a reason. The researcher conducted the interviews in Arabic and English according to each participant’s preference. Each interview lasted between 15 and 30 min. Interviews were audio-recorded with the permission of participants. For participants who declined to have an audio-recorded interview, only researcher filed notes were taken. Data collection continued to data saturation, until the emergence of no new issues.

### Translation of the interview schedule

The questionnaire was translated and validated through a three-stage process of instrument validation including translation, group validation and post-validation [14]. The interview schedule was translated using a parallel blind approach which included two bilingual researchers independently translating the instrument from the original language (i.e. English) into the target language (i.e. Arabic). Both translators were proficient in English and native speakers of Arabia, and had experience in research interviewing and health-related qualifications, as well as being familiar and professionally trained with the concepts being examined in the current study. The two translated drafts were then compared to see which words and phrases best convey the precise meaning and complexity of the original questionnaire, and any deviation addressed. The tool was further reviewed by an Arabic-speaking researcher, pharmacists and a group of target language speakers (*n* = 3) who were asked to comment on the language, content and cultural sensitivity of the tool.

### Data processing, analysis and quality assurance

A similar process of interview translation undertaken in Arabic was conducted in parallel by two bilingual speakers independently following data collection. Any discrepancies were discussed and resolved. Data analysis and transcription of interviews were on-going through the data collection process to enable a judgement to be reached regarding data saturation and the need for no further information or data.

The interviews were analysed thematically using an inductive approach and NVivo 10 software. The inductive approach was used to allow patterns and themes to generate and emerge by grouping descriptions from open (unstructured) questions. Therefore, a primary coding structure was developed by the research team by using a subset of data which reflected participants’ experiences and description of issues and reasons behind self-medication practice. Three members of the research team and one researcher external to the study derived the primary codes independently. The results were compared and discussed, and discrepancies were resolved by discussion.

## Results

Forty participants (of a total of 45 invited to do so) took part in the interview (response rate: 89%). A full overview of the participants’ characteristics is shown in Table 1. The frequency of participants’ self-medication with antibiotics in the past 2 years was high: Very often (≥4 times: 23, 58%); Often (3 times: 5, 12%); Sometimes (2 times: 7, 18%); and Rarely (1 time: (5, 12%) and it was associated with various inappropriate antibiotic use practices and negative outcomes such as antibiotic resistance, treatment failures and adverse events. Interviews revealed that different reasons contribute to the rise of self-medication with antibiotics, ranging from difficulty accessing healthcare services, participant’s cultural beliefs and practices, lack of knowledge/awareness about antibiotics and antibiotic resistance, and weak regulatory enforcement. Table 2 lists all themes/subthemes that emerged from interviews in relation to factors contributing to self-medication with antibiotics from participants’ perspectives. Each of these reasons is discussed in more detail below, with illustrative quotes from the participants’ transcripts.

**Table 1:** Characteristics of participants recruited into the study

Parameter		Results
Age (years)	Mean (SD)	30 (10.2)
Gender [N (%)]	Male	8 (20)
	Female	32 (80)
Nationality [N (%)]	Saudi	40 (100)
Living arrangement [N (%)]	Family	38 (95)
	Alone	2 (5)
Education [N (%)]	> High school	35 (88)
	≤ High school	5 (12)
Health insurance [N (%)]	Yes	24 (60)
	No	16 (40)
Income per month [N (%)]^a^	> 4266 USD	7 (17)
	2133 – 4266 USD	15 (38)
	< 2133 USD	18 (45)

^a^One United States Dollar (USD) = 3.75 Saudi Arabian Riyal (SAR) [Date of change 30/08/2018]

**Table 2:** Themes/subthemes that emerged from interviews in relation to factors contributing to self-medication with antibiotics from participants’ perspectives

Category	Themes/subthemes identified within this category
Factors contributing to self-medication with antibiotics from participants’ perspectives	Participants’ cultural beliefs and practices regarding antibiotics • Cultural perception regarding the curative power of antibiotics • Storing antibiotics at home for later use and sharing them with family members • Previous experience with antibiotics and similar symptoms
	Lack of knowledge/awareness about antibiotics and antibiotic resistance
	Problems attributed to access to, and organisation of, services • Difficulty getting appointments and long waiting time during visits • Perception about practitioners and less shared decision making • Transportation issues • Weak regulatory enforcement mechanism that prevents the selling of antibiotics without prescriptions • Cost of care • Stigma of getting an infection upon going to the hospital for a consultation

### Factors contributing to self-medication with antibiotics from participants’ perspectives

#### Participants’ cultural beliefs and practices regarding antibiotics

##### Cultural perception regarding the curative power of antibiotics

Some participants believed that antibiotics can speed-up recovery and treat and eradicate any infection. One participant discussed her experience with antibiotics and stressed their efficacy in helping her to “get better quickly”, as illustrated in the following quote:

“Antibiotics kill things very quickly, take [them] and in two days you will be fine.” [P5].

Another participant assumed that the power of antibiotics could deal with all her health problems:

“Augmentin can be used for everything.” [P8].

This belief led some participants to take antibiotics with them when travelling abroad “just in case” they or their family get sick:

“I take Augmentin with me when I travel just in case me or my family get sick [laughs].” [P11].

For one participant, it was particularly interesting to find that he completely believed that antibiotics increase a person’s immunity and are effective in providing immediate relief:

“It’s a 100% immunity booster and can provide quick relief.” [P25].

Another participant said that she always considered packing a short course of antibiotics in order to treat or prevent any possible infection that could ruin her holiday, especially when visiting countries that do not allow travellers to buy antibiotics without a prescription:

“When I travel, [in case] I get sick, especially in countries that do not allow people to buy antibiotics without a prescription, I bring along with me from home an antibiotic which has a short-course duration to help me recover quickly.” [P12].

Having a strong belief in the curative power of antibiotics had led one participant to use a short course of antibiotics during the holy month of Ramadan when she became ill, since this approach enabled her to recover quickly without the need to break her fasting:

“When I encounter an infection which requires an antibiotic during the holy month of Ramadan, I use Azithromycin [as] this will help me to recover quickly and continue my fasting.” [P12].

An interesting cultural belief which emerged related to certain brands of antibiotics having a better quality and more curative power than others. For example, some participants believed that Western brands of antibiotics were somehow more effective at relieving symptoms than the local alternatives, which in the end led to inappropriate use of antibiotics:

“I got flu while travelling and I bought some Augmentin. I used it for 10 – 14 days without benefit because it was manufactured in the United Arab Emirates and it was not original [it was generic]. When I came back to my country, I went to buy original Augmentin from the pharmacy.” [P1].

##### Storing antibiotics at home for later use and sharing them with family members

People wanting to store antibiotics at home for later use and sharing them with family members were common practices among study participants. Sharing of antibiotics allowed people to demonstrate care for one another and tackle the issue of sickness as a unit. It also allowed them to bypass accessing healthcare services in an effort to obtain treatment for common problems. Participants indicated that they shared antibiotics usually with close family members and less usually with neighbours, friends and work colleagues. Participants seemed to recognise that buying antibiotics without a medical consultation was against Saudi law, but, because the behaviour was consistent with Saudi culture, they did not seem troubled by this. Two participants made the following observations in relation to this:

“I had flu and the doctor gave me an antibiotic and it worked for me. Then, I went back and recommended it to my family. It is not only me, even my mother tried an antibiotic two years ago and she found it useful, and then she advised the whole family to use it.” [P36].

“No need to see a doctor over a minor thing that I experienced before. Well, I can’t remember exactly what the antibiotic is but my whole family use it and [it] is always available at home; its’ a big box with white and purple colours [Augmentin].” [P24].

##### Previous experience with antibiotics and similar symptoms

The third sub-theme to emerge was related to participants’ previous success with antibiotics. When they experienced symptoms that they had experienced before, they sought the treatment that had been successful previously. The participants reported that they had usually sought medical advice from a healthcare provider, often a doctor, for the first episode of a specific illness. If they had a subsequent episode with a similar symptom complex, they felt comfortable with a self-diagnosis. Therefore, because they are relying on previous experience with similar symptoms to identify the condition, diagnosis and treatment, self-medication seems appropriate, a behaviour which may have substantial public health implications. This is illustrated in the following scenarios:

“I used to go to the doctor but later I treat myself by my own experience. Let’s say that I went to a doctor once and he gave me an antibiotic and I found it effective, I use it again if needed. No need to go to the doctor again.” [P3].

“It is pink but I don’t remember the name. The doctor a long time ago gave it to me and I started to buy it from the pharmacy whenever I have a sore throat.” [P23].

Such practice has led to the development of intolerable side effects and allergies among some of the study participants, which they reacted to and dealt with in different ways – by early discontinuation of antibiotics before completing the course or by modifying the way they took their antibiotics. These reactions were always self-guided:

“I remember that I bought Augmentin over the counter and [it] caused an allergy for my son and I stopped it immediately.” [P5].

“Once, I used an antibiotic and I had a fast heart rate after taking it. Well, I [was] supposed to take it for seven days but I used it only for five days.” [P2].

Fear of side effects had led some of the participants to deliberately take less of their antibiotics without consulting a doctor, in order to avoid these adverse effects. One participant declared:

“If I used the antibiotics without a prescription and it is written that you should take three tablets, I used to take two tablets to be on the safe side and avoid the side effects.” [P16].

Another two participants reported that they tend to use topical antibiotics to avoid side effects because they believed that applying antibiotics to the skin does not cause as many side effects as swallowing antibiotics that circulate through the body. One participant stated:

“Once I took a topical antibiotic for my skin infection; I used it all the time. I thought that because it is topical would have [fewer] side effects than oral. So, it would be OK if I used it as much as I wanted.” [P13].

Stopping antibiotics when feeling better or as soon as symptoms disappeared was also a common practice among some study participants in order to avoid side effects. One participant said:

“Once I feel better, I stop the antibiotic although the person has to take the whole course.” [P8].

#### Lack of knowledge/awareness about antibiotics and antibiotic resistance

Based on the knowledge part of the questionnaire (i.e. section number 2) that assesses knowledge about antibiotics and antibiotic resistance, 60% (24/40) of participants reported sub-optimal knowledge (i.e. where intermediate and poor knowledge were combined) of antibiotics. More than half of the participants (22/40, 55%) had misconceptions about antibiotics as they believed that they are effective in treating viral infections. One participant voiced:

“Antibiotics are medicines that are used to treat different infections. Some are used to treat influenza and other are used to treat infections, etc.” [P21].

A majority of participants (30/40, 75%) held misconceptions that antibiotics can be purchased and taken without a doctor’s prescription. Thirty-eight percent (15/40) of participants incorrectly believed that it is safe to switch between antibiotics during the course of a single infectious disease. Fifty-five percent of participants (22/40) did not know that they should not stop taking a course of antibiotics as soon as they feel better. One participant declared:

“I sometimes don’t take the full treatment course; for example, if the treatment is for five days, I take the antibiotic for three days and then I stop it once I feel better.” [P21].

One-third (12/40) of participants thought that it is fine to take a missed antibiotic dose with the next dose. Others (10/40, 25%) incorrectly believed that taking many types of antibiotics at the same time during the course of a single illness will result in quick recovery. Forty percent of participants (16/40) held the misconception that antibiotics are the same as anti-inflammatories.

Many participants (16/40, 40%) were not very sure about what antibiotic resistance is, nor its causes or consequences, when they were asked what they know about antibiotic resistance. Some (13/40, 33%) thought that over-use of antibiotics might be the cause of antibiotic resistance and reduction of their future effectiveness. Others (11/40, 27%) believed that bacterial resistance would be increased when using antibiotics not according to the doctor’s advice. One participant mistakenly believed that using the same antibiotic again for flu symptoms would increase antibiotic resistance:

“We should not repeat using the same antibiotic again if we experience another [case of] flu, because our body will get used to it, so we have to change it.” [P22].

Another participant thought that changing from one antibiotic to another or reducing the strength of an antibiotic will help in reducing antibiotic resistance:

“I know that, if you do not take [an] antibiotic as instructed, you might develop antibiotic resistance. So, what you have to do is either you change [the] antibiotic to another one or you reduce the strength of [the] antibiotic, say from 500 to 250 mg.” [P37].

#### Problems attributed to access to, and organisation of, services

##### Difficulty getting appointments and long waiting time during visits

Long waiting time for consultation and difficulty getting appointments were barriers weighted against the benefits of visiting a doctor for these seemingly uncomplicated and common infections. The participants reported that they would consult a physician in case of emergency situations or critical conditions. Some participants noted that they called family members who were healthcare professionals to provide an expert opinion when they had concerns about the accuracy of their self-diagnosis. This was still seen as a less-time consuming and easier approach than consulting a physician, as outlined in the following quotes:

“When you go to the government hospitals, you need a long time to see a doctor, either waiting during or waiting for the appointment. Sometimes, [it takes] one or two months to see a doctor.” [P3].

“My father is a pharmacist so, when I have an infection, I do consult my father. It is faster and less time consuming than seeing a doctor.” [P1].

##### Perception about practitioners and less shared decision making

Some participants perceived their doctors to take full charge during the consultation process, making the ultimate decision about therapy without explaining the process or considering the views/preferences of the patient, which led them to self-medicate. This is illustrated in the following scenario:

“The doctor doesn’t say much; he would only prescribe the medicine and that’s it! He does not look at my medical file. Even when I try to discuss something with him, he just doesn’t care or pay attention. So, my family and I decided to go directly to the pharmacy and get what we need from there.” [P13].

##### Transportation issues

Some women reported that they could not access healthcare services easily to consult a doctor due to transportation issues because, until recently, women in Saudi were not allowed to drive. One participant reported that she usually depends on her husband to bring a certain antibiotic for her:

“I cannot remember the name of the antibiotic, but we always use it. My husband usually brings it for me since he has the experience and he has the car as well [laughs]. I just tell him to bring the antibiotic from the pharmacy and he brings it for me.” [P6].

Another participant said that she used to buy antibiotics from the pharmacy since the hospital is far away from her home and she might not always have proper transportation to go there:

“Since I live in Qatif and the hospital is in Khobar and I don’t have transportation, I usually buy the antibiotic from the pharmacy that is beside my home.” [P9].

##### Weak regulatory enforcement mechanism that prevents the selling of antibiotics without prescriptions

Some participants indicated that, since antibiotics are available and easily accessible in the pharmacies, people will buy them directly from there without consulting a doctor, because the enforcement of regulations is weak and antibiotics are often sold without a prescription:

“Since it is readily available in pharmacies and once you request it, you receive it just like in a grocery store, I will buy it from the pharmacy or I will just send my driver to bring it.” [P19].

“They are available everywhere and you can get them easily. The law is not strictly enforced to prevent [the] selling [of] antibiotics without prescriptions.” [P23].

For one participant, it was particularly interesting to find that a pharmacist encouraged her to get an antibiotic from his pharmacy without prescription, despite the fact that she did not need it, just because she has insurance – as illustrated in the following quote:

“I said, ‘If I’m feeling OK, why do I have to get an antibiotic from the pharmacy?’ He said, ‘Why not? You have insurance; you can keep the antibiotic with you just in case you need it for later use.’ I was really surprised when he said this.” [P28].

##### Cost of care

Some participants declared that one of the reasons influencing people to self-medicate with antibiotics is low economic status and lack of health insurance, which might be contributing further to the difficulty in accessing the healthcare system. They explained that people lacking health insurance or those who have a low income tend to buy antibiotics directly from community pharmacies to avoid the trouble and costs of visiting a doctor, although this might be a burden on their budgets. They observed that the cost of a hospital visit is more expensive than that of an antibiotic, although the antibiotics are not cheap:

“I’m not going to go to the physician, if I already know my symptoms and what medicine heals me. If I go, they will charge me just for the consultation, and then I have to buy the prescription and that’s another cost with more money. It is easier and cheaper to go and buy an antibiotic straightway, if I know what medication I’m going to take and will work for me.” [P12].

“I think, nowadays, most people go to self-medication either because they don’t have health insurance, and they have to pay money sometimes that they cannot afford, and sometimes it is a burden on their budget and so they self-medicate.” [P3].

##### Stigma of getting an infection upon going to the hospital for a consultation

Another reason which reflects a cultural norm and contributes to self-medication with antibiotics is the stigma of getting an infection upon going to hospital, making people less likely to access the healthcare system. This was mentioned by one of the participants who believed that a person might develop a more severe infection when going to a hospital, which enhances her tendency to self-medicate with antibiotics:

“Once [upon] a time, there was a period when you got an infection such as corona etc. upon going to the hospital, which enhances my desire to avoid visiting a doctor and getting an avoidable infection.” [P7].

## Discussion

This article presents the first study exploring a wide range of factors influencing self-medication practice, and providing a more inclusive picture of such practices and the circumstances in which they arise, providing novel evidence that SMA among this particular population is problematic in Saudi Arabia. Only four other studies have addressed the problem of SMA in Saudi Arabia [15–18]; however, none of them included the people’s perspective regarding SMA using an in-depth interview technique.

In this study, the frequency of SMA among participants was high and it was associated with various inappropriate antibiotic use behaviours such as altering or stopping antibiotic-taking, either when feeling better (to relieve the body) or when feeling worse (to alleviate adverse effects). This shows that customers are not passive recipients of care. Rather, they sometimes develop their own way of taking their antibiotics based on their circumstances or what makes sense to them. Thus, assessing participants’ beliefs, fears and expectations and exploring their practices regarding antibiotic use are necessary to prevent irrational use of antibiotics.

An exploration of factors influencing self-medication behaviour among study participants revealed many similarities with evidence from the Middle Eastern literature [3], although some differences were also found which may appear to be specific to the Saudi population. For example, in line with the Middle Eastern literature, lack of a strong regulatory enforcement mechanism to improve rational use of antibiotics, and gaps in terms of knowledge, attitudes and practices regarding antibiotic use – such as keeping leftover antibiotics for future use, sharing antibiotics with others, self-diagnosis and people’s desire to buy antibiotics in sub-therapeutic quantities in order to treat illness or not to become ill, belief that antibiotics can speed up recovery and eradicate any infection – were all found to be possible reasons for SMA. Past successful experience with similar illness and knowledge of the drugs that were prescribed previously, low economic status, lack of health insurance and lack of access to healthcare were also reasons contributing to the rise of SMA.

In terms of reasons for SMA specific to this particular population, the current study identified some beliefs and practices about antibiotic use that have not been adequately described in the literature to date, such as beliefs participants held about Western brands of antibiotics and topical antibiotics, taking antibiotics while travelling abroad or being away from home for ‘just in case’ purposes, and using a short course of antibiotics during the holy month of Ramadan or while travelling abroad to enable quick recovery. In addition to these beliefs and practices, the stigma of getting an infection upon going to hospital to consult a doctor, and perceptions about healthcare providers and healthcare system, such as paternalism and ignoring the patient’s perspective during medical consultations, issues relating to the organisation of the healthcare system and the impact of this on access to healthcare services or antibiotic supply appeared to be findings specific to the Saudi population. If not addressed, these factors may lead to unanticipated adverse events, complications of incorrect use or misuse, delay in seeking professional help and antibiotic resistance. By uncovering factors influencing self-medication behaviour, the study can provide insight when designing future interventions to promote safe, rational and effective use of antibiotics.

Strengthening the role of pharmacists from traditional drug dispenser to more effective healthcare provider is required to improve the rational use of antibiotics. Pharmacists are the key healthcare providers with the training, skills and knowledge associated with the profession required to minimise self-medication behaviours. Thus, it is vital to recognise and use their potential. Pharmacists in many European countries already have the ability to take on additional responsibilities and roles to promote the rational use of antibiotics. For example, many new forms of rapid diagnostics/point-of-care testing (PoCT) are being developed that will give simple-to-use and inexpensive approaches of deoxyribonucleic acid/ribonucleic acid (DNA/RNA)-based recognition of potentially multiple pathogens from a single sample [19]. These new forms of PoCT can make it possible to provide the correct treatment immediately and foster antimicrobial stewardship and tackle antibiotic resistance, when they become available. Antibiotic stewardship is defined as promoting the appropriate selection, dose, duration and route of administration of antibiotics [19–21]. In the United Kingdom, some pharmacies in London are conducting PoCT linked to sore throat and, if bacterial tonsillitis infection is identified, are supplying penicillin-V (or, clarithromycin in case of allergy) [19–21]. This is made possible through pharmacy-based patient group direction (PGD). PGDs provide a legal framework that permits the supply and/or administration of specified medicine(s), by certain registered healthcare providers, to a pre-defined group of patients, without the need for an instruction or a prescription from a provider [19, 22].

This study provided evidence that 60% of participants had insufficient knowledge about antibiotics. There was also no or little recognition of the impact of SMA on antibiotic resistance. The insufficient knowledge about antibiotics could be translated into various inappropriate antibiotic use practices and negative outcomes such as antibiotic resistance, treatment failures and toxicity. Studies from the Middle Eastern literature have reported similar knowledge deficits among Middle Eastern populations [3]. The insufficient knowledge about antibiotics and antibiotic resistance among participants could be related to the lack of patient education and counselling provided by healthcare providers. It has been argued that knowledge is a prerequisite of preventive health behaviour and can motivate patients to take an active role in the treatment of their disease [23].

Since patients frequently visit pharmacies to obtain antibiotics for self-medication, pharmacists are well placed to improve rational use of antibiotics among pharmacy customers. The community pharmacists should take an active role in different public-health initiatives relating, for instance, to the restriction of irrational dispensing of antibiotics and enhancing public awareness of the importance of stopping self-medication without correct diagnosis and of the rising issue of antibiotic resistance infection in the society [19, 24]. With patient counselling, pharmacists are also in a great position to identify and correct any false beliefs patients might have and address any concerns they might have, in order to make sure they have a better understanding of the rational use of antibiotics.

Since antibiotics in Middle Eastern countries can be obtained without prescription, research on effective strategies to limit unnecessary antibiotic use should target doctors’, pharmacists’ and patients’ knowledge and behaviours. Thus, addressing the knowledge gap and implementing multifaceted behaviour change interventions are likely to be effective. Malta, for example, has introduced a European Antibiotic Awareness Day [25] in an attempt to increase knowledge and awareness among the Maltese public, prescribers and pharmacists, and ensure that regulations are enforced. Consequently, self-medication has fallen from 19% of Maltese respondents admitting taking antibiotics without a prescription in 2001 to 2% in 2016 [26, 27]. While awareness campaigns and education are often recommended [28, 29], interventions targeted specifically at changing behaviour are more likely to be effective.

The key to successful strategies for managing antibiotic resistance is to promote behaviour modification besides providing relevant information on proper antibiotic use [30]. Behavioural theories, such as the theory of planned behaviour [31] and the stages-of-change model [32], and social science methods have been suggested as suitable approaches to better understand factors influencing prescribing practices [33] but no behavioural theories have been suggested or developed in order to understand factors that influence self-medication behaviour among the general public.

A well-studied model that can be used to explain or predict behaviour change is the Transtheoretical Model (TTM) [34]. A wide variety of target health behaviours have been studied using the TTM paradigm, including smoking cessation, weight control, exercise, stress management, alcohol and drug abuse, screening recommendations adherence, and medication management [35]. The utility of the TTM for the pharmacist is to recognise the stage of behavioural change the patient is currently in, and then use the associated stage-matched tools to help the patient move towards the next stage. The five primary stages of change are:

1. Pre-contemplation: where an individual is unaware that his/her current behaviour (e.g. SMA) constitutes a problem and thus has no intention of changing it.
2. Contemplation: the individual is thinking about changing the risky behaviour but is not yet committed.
3. Preparation: the individual has an intention to change the behaviour and is starting to make plans about how to change it.
4. Action: the individual is actually attempting to change the behaviour.
5. Maintenance: the individual is six months abstinent from the risky behaviour and is attempting to prevent relapse.

After classifying a patient into one of these stages, the pharmacist can develop appropriate interventions to assist the patient in moving towards the next stage. Individuals may cycle through the stages several times before achieving long-term behaviour change. Rewards, reminders and monitoring techniques may be needed for individuals in later stages of behaviour change, but, for patients in earlier stages, discussion of available OTC alternatives and consciousness raising that focuses on the disadvantages of SMA and advantages of stopping this ‘habit’ are needed. However, the application of TTM to influence self-medication practice among the general public is limited, and its efficacy within this context remains unclear. When applied correctly, TTM may provide the necessary approach to promote sustained behaviour change among targeted populations.

This study also detected the important role of patients’ families in their infection management in general and in antibiotic-taking in particular. Families were frequently quoted as an important source of support, providing advice and supply of antibiotics to participants. In line with published studies in Kuwaiti culture [36], Saudi people value family intimacy and have the advantage of cohesive and supportive family networks. Healthcare providers need to be aware of this, as some patients may obtain false or inaccurate information or advice from their families and initiate changes in their antibiotic intake or take inaccurate antibiotics accordingly. In this study, all participants were living with their families. This suggests that a family-centred approach to education by healthcare providers may also be beneficial. It has been reported that personal interaction in the form of counselling or group sessions might be more successful than simply handing out written material; such group sessions may be useful as a reference or refresher of patients’ knowledge but should not replace ongoing patient education and counselling that should be provided by healthcare providers (HCPs).

Law enforcement and stricter governmental policy regarding the sale of antibiotics without prescription in retail pharmacies and irrational antibiotic usage are also needed. In Chile, for example, after drawing up prescription-only regulations, consumption of oral antibiotics in the community pharmacies remarkably decreased [37]. In Saudi Arabia, prescription-only regulations are embedded in the Drug Law. However, there is no penalty for non-compliance, despite these regulations. This may explain why, to date, no pharmacy has been penalised for antibiotic dispensing without medical prescription. Self-medication is viewed as more economical and convenient than visiting a HCP, as there is a lack of enforcement of the regulations.

Robust antimicrobial use surveillance systems are essential to combat antibiotic resistance. In most European countries, there is a long tradition of monitoring the use of medicines but this should also be implemented in Middle Eastern countries. Antimicrobial stewardship courses in the pharmaceutical and medical postgraduate and undergraduate curricula are also required. The information provided in these courses should stress the importance of the roles of the pharmaceutical and medical professions in fostering the rational use of antibiotics.

### Strengths and limitations

Strengths: (1) this article has presented the first study exploring a wide range of factors affecting self-medication practice, and providing a more comprehensive picture of such practices and the circumstance in which they arise. Limitations: (1) the sample of this research comprised Saudi participants living in the Eastern Province of Saudi Arabia; therefore, results may not be transferrable to the whole country, or to non-Saudis; (2) this study only involved the general public or community members and did not include other suppliers of antibiotics, such as pharmacists and doctors; (3) some participants were a little anxious about being recorded. This was apparent with a few participants who were anxious about the digital recorder for cultural reasons as they were not sure who would be listening to their voice. However, the researcher reassured them that she was the only one who would listen to the recording, and explained to them that data would be anonymous and confidential, and that it would be destroyed upon completion of the study. Once participants realised this, they were happy for the interview to be recorded. It is worth noting that, for future research purposes, careful attention must be paid to this issue as some patients may find it intimidating to be recorded and may not feel free to provide their honest views in such circumstances.

### Implications for future research

Further studies are needed to design, implement and then evaluate culturally sensitive and effective interventions that are tailored to the target audience in whom behaviour change is required in order to decrease antibiotic self-medication practices. Currently, there are no published results of interventions to prevent this practice, which has important implications for public health and the development of antibiotic resistance [38]. This can lead to an understanding of the facilitators of and barriers to behaviour change, enable designing of interventions that overcome barriers and utilise facilitators, to provide more effective and sustainable outcomes. Alternatively, future research could focus on self-medication from public and healthcare providers’ perspectives (physicians and pharmacists) using a triangulation technique to provide an in-depth exploration and more comprehensive understanding of the factors that influence self-medication.

## Conclusions

The study indicates that SMA is common practice in Saudi Arabia. By uncovering factors influencing self-medication behaviour among the public, the study can provide insight when developing future interventions to improve prudent, safe and effective use of antibiotics.

## Additional file

Additional file 1: Interview schedule or interview guide. (DOCX 63 kb)

## Abbreviations

CDC: Centers for Disease Control and Prevention
DNA/RNA: Deoxyribonucleic acid/ribonucleic acid
MOH: Ministry of Health
MRSA: Methicillin-resistant *Staphylococcus aureus*
OTC: Over-the-counter
PGD: Patient group direction
PoCT: Point-of-care testing
SAR: Saudi Arabian Riyal
SMA: Self-medication with antibiotics
TTM: Transtheoretical Model
USD: United States Dollar
WHO: World Health Organisation
