# Supplementary material for: “Antibiotics kill things very quickly” - consumers’ perspectives on non-prescribed antibiotic use in Saudi Arabia
Source: BMC Public Health. 2018 Oct 16;18:1177. doi: 10.1186/s12889-018-6088-z (PMC6192199; doi:10.1186/s12889-018-6088-z)
Supplement: Supplementary file 1 — Interview schedule or interview guide. (DOCX 63 kb) [file 12889_2018_6088_MOESM1_ESM.docx]

**Additional file 1: Interview schedule on self-medication with antibiotics**

Part 1: Participants’ characteristics

| Items | |
| --- | --- |
| 1. Age |  |
| 2. Gender | Male |
|  | Female |
| 3. Nationality | Saudi |
|  | Non-Saudi |
| 4. Educational level | > High school |
|  | ≤ High school |
| 5. Living arrangement | Family/relatives |
|  | Friends |
|  | Alone |
|  | Other, please specify ………. |
| 6. Income per month | > 4,266 USD |
|  | 2,133 – 4,266 USD |
|  | < 2,133 USD |
| 7. Health insurance | Yes |
|  | No |
| 8. How would you rate your general health status | Very good |
|  | Quite good |
|  | Neither good nor poor |
|  | Quite poor |
|  | Very poor |

Part 2: Knowledge about antibiotics

| Items | YES | NO |
| --- | --- | --- |
| 1. Antibiotics can be purchased and taken without a doctor’s prescription. |  |  |
| 2. It is OK to switch between antibiotics during the course of single infectious disease. |  |  |
| 3. Missed doses of antibiotic can be taken with the next dose. |  |  |
| 4. Taking many types of antibiotics at the same time during the course of a single illness will result in quick recovery. |  |  |
| 5. You can stop treatment as soon as the symptoms had disappeared. |  |  |
| 6. Antibiotics are the same as anti-inflammatories |  |  |
| 7. Antibiotics are effective in treating viral infections |  |  |
| 8. People can be allergic to antibiotics. |  |  |
| 9. Antibiotic overuse can result in antibiotic resistance |  |  |
| 10. Bacterial resistance would be increased when using the medication not according to the doctor advice for many times |  |  |

Part 3: Participants’ views regarding self-medication with antibiotics

| Items |
| --- |
| 1. What do you know about antibiotics? |
| 2. From where do you obtain antibiotics?  *(Prompt: Pharmacy/drug shop, Family members/friends, Left over from prior use, etc.)* |
| 3. Do you take a full course of antibiotics?  Have you had examples when you didn’t? |
| 4. How do you often self-medicate with antibiotics?  When was the last time? Why?  With what antibiotic?  And for what conditions? |
| 5. From who/where do you get information on self-medication with antibiotics?  *(Prompt: Family, Doctor, Pharmacist, Old prescription, Leaflets, Own decision, Media (TV, magazines and internet), Friends/neighbours, academic knowledge, Books)*  What information do you get on self-medication with antibiotics?  *(Prompt: When to take the antibiotic, how to take it, etc.)* |
| 6. What do you know about antibiotic resistance? |

***Thank you very much for your participation in this interview***
